# Prospective applications of two-dimensional materials beyond laboratory frontiers: A review

**DOI:** 10.1016/j.isci.2023.106671

**Published:** 2023-04-14

**Authors:** Partha Kumbhakar, Jitha S. Jayan, Athira Sreedevi Madhavikutty, P.R. Sreeram, Appukuttan Saritha, Taichi Ito, Chandra Sekhar Tiwary

**Affiliations:** 1 Metallurgical and Materials Engineering, Indian Institute of Technology, Kharagpur, West Bengal 721302 India; 2 Department of Physics and Electronics, CHRIST (Deemed to Be University), Bangalore 560029, India; 3 Department of Chemistry, National Institute of Technology Calicut, Calicut, Kerala, India; 4 Department of Chemistry, Amrita Vishwa Vidyapeetham, Amritapuri, Kollam, Kerala, India; 5 Department of Chemical System Engineering, The University of Tokyo, Tokyo 113-0033, Japan; 6 Center for Disease Biology and Integrative Medicine, Faculty of Medicine, The University of Tokyo, Tokyo 113-0033, Japan

**Keywords:** Materials science, Nanomaterials, Materials application

## Abstract

The development of nanotechnology has been advancing for decades and gained acceleration in the 21st century. Two-dimensional (2D) materials are widely available, giving them a wide range of material platforms for technological study and the advancement of atomic-level applications. The design and application of 2D materials are discussed in this review. In order to evaluate the performance of 2D materials, which might lead to greater applications benefiting the electrical and electronics sectors as well as society, the future paradigm of 2D materials needs to be visualized. The development of 2D hybrid materials with better characteristics that will help industry and society at large is anticipated to result from intensive research in 2D materials. This enhanced evaluation might open new opportunities for the synthesis of 2D materials and the creation of devices that are more effective than traditional ones in various sectors of application.

## Graphical abstract

**Figure:**
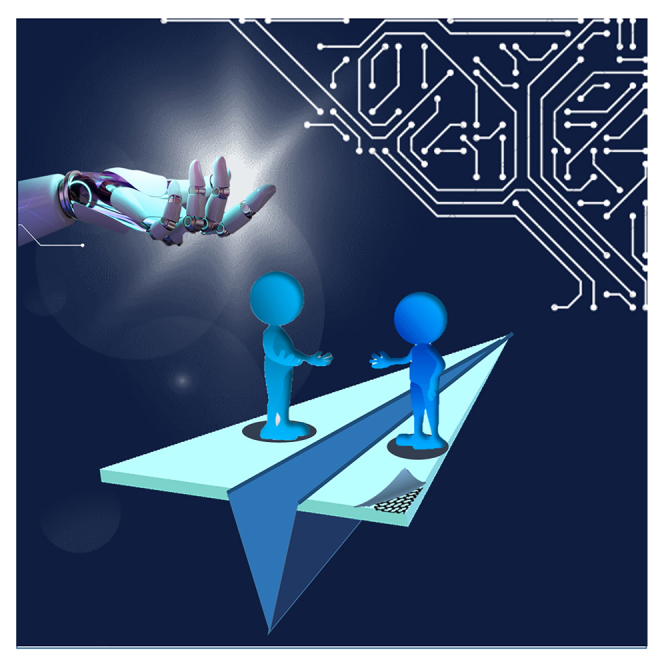

## Introduction

The continual improvements in science and technology have enabled the development of new scientific inventions and discoveries in various aspects. In the era of scientific inventions, atomic-level materials have been extensively studied for the development of outstanding materials that improve the elemental output in a variety of applications. In the present century, advancement in nanotechnology is changing the world’s outlook in different ways. Nanotechnology has made a revolution in technology and has brought tremendous growth in the field of medicine, materials, electronics, and others. Single-atom-thick materials have higher strength compared to 3D materials as well as a higher surface area to volume ratio which increases their rate of reactions.^1^

**Figure 1:**
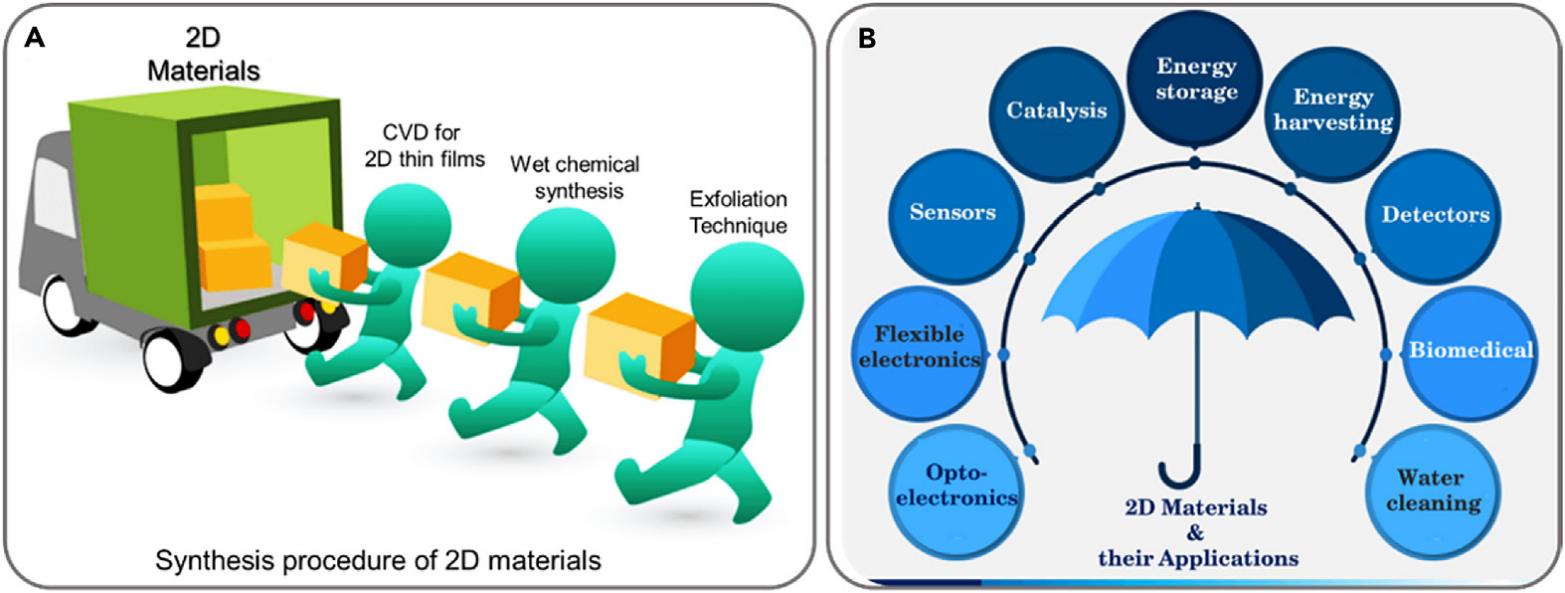
Overview of the synthesis method and applications (A) Schematic representation of synthesis techniques of 2D materials. (B) shows the representation of applications of 2D materials on both the academic and industrial scenes.

The interdisciplinary methods that combine inventive processing with molecular engineering allow scientists to create complex systems with a flawless command of various size scales, compositions, functions, and morphologies.^2^ Cross-cutting synthetic strategies are required to create hierarchical hybrid architectures, which opens up a world of possibilities for the discovery of new materials with potential applications in a variety of industries, including the environment, energy, housing, health, automotive, micro-optics, and micro-electronics. As seen by the few instances depicted in the Figure 1, a first emergence of 2D material applications has already taken place today. Smart membranes and coatings, photovoltaic/fuel cells, therapeutic biosensors, catalysts, textiles, and automotive parts provide us a good but incomplete view of the current material items, components, or systems. The 2D materials, whose development has only entered its adolescence, are incredibly promising advanced materials. Without a doubt, 2D materials created through green chemistry will produce an increasing number of smart membranes, new catalysts and sensors, advanced generations of photovoltaics and fuel cells, smart micro-electronics, micro-optical and photonic components and systems, or intelligent therapeutic vectors that combine targeting, imaging, and therapy.

Here, we provide a quick summary of current developments in 2D crystals and their numerous uses in a variety of industries, including energy production, energy harvesting, composite coating, biological applications, etc. After the discovery of graphene, 2D materials have attracted significant attention, and its numerous synthesis techniques have been developed (Figure 1A). Top-down and bottom-up techniques are two major categories that may be used to synthesis processes for 2D materials. The top-down method, which entails cleaving atomically thin layers from bulk crystals, is mostly utilized for layered materials. The two approaches that are most frequently used to separate layers of materials are mechanical forces and acoustic vibrations. While obtaining 2D layers via the top-down methods including mechanical and chemical exfoliation have emerged as the preferred options. Molecular beam epitaxy, chemical vapor deposition, pulsed laser deposition (PLD), and other solution-based techniques are examples of bottom-up techniques that may be used to create 2D structures on a massive scale. We next comprehensively analyze the numerous uses of the 2D materials via structural control based on how to modify their structure, components, and electronic state in order to maximize performance (Figure 1B). Finally, we provide a view on future research possibilities and difficulties in relation to both the conceptual understanding and practical use of such 2D materials.

## Applications of 2D materials in both the academic and industrial scenes

### Energy conversion, storage, and thermoelectric property

**Figure 2:**
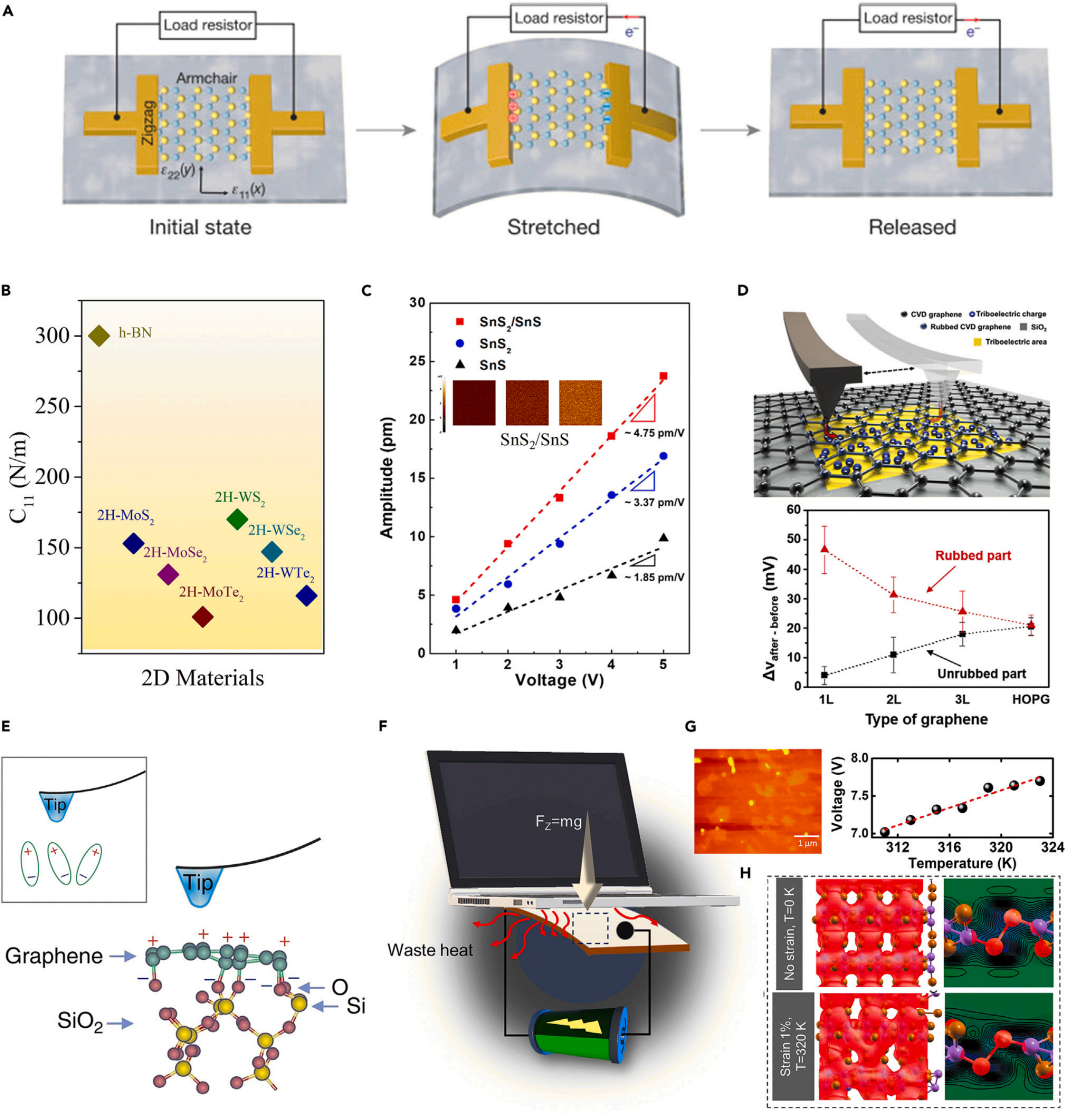
Energy harvesting from 2D materials (A) The piezoelectric device with single-layer of MoS_2_ and its piezoelectric mechanism.^6^ (reproduced with permission from Nature). (B) Summary of the piezoelectric coefficient of 2D materials.^7^ (reproduced with permission from American Chemical Society). (C) Images of the piezoelectric response amplitude in response to applied voltages between 1 and 5 V. Effective piezoelectric coefficients of SnS, SnS_2_, and SnS_2_/SnS heterostructure thin films^4^ (reproduced with permission from American Chemical Society). (D) Schematic of the friction process and the kelvin probe force microscopy measurement system (Upper panel). Potential difference for different types of CVD graphene (1, 2, or 3 layers) and for HOPG.^8^ (reproduced with permission from Nature). (E) Schematic diagram of a graphene layer on a SiO_2_ substrate with an O terminal and dipoles being created by the chemical reaction between the atoms of C and O.^5^ (reproduced with permission from Nature). (F) Diagram illustrating the concept of piezo-tribo generators utilizing 2D CoTe_2_ to harvest energy and use waste heat to generate power. 2D CoTe_2_ AFM image (right upper). (G) Output voltage vs. temperature fitted linearly. (H) The electron density map at right from the side view at 0 K, no strain, (upper panel) and 320 K, 1% strain (lower panel), and the electron density plot^9^ (reproduced with permission from Royal Society of Chemistry).

The study of 2D materials has recently gained attention due to its potential uses in several industries, including optoelectronics, spintronics, sensors, thermoelectric, photoelectric, superconductors, energy storage, and topological insulator devices.^1^^,^^2^^,^^3^^,^^4^^,^^5^^,^^6^^,^^7^^,^^8^^,^^9^ Due to their intriguing physicochemical characteristics and potential applications in optoelectronics, semiconductor technology, energy storage, battery electrodes, superconductivity, electrocatalysis, thermoelectricity, and gas sensing, 2D metal chalcogenides have received a great deal of attention in research. Due to their exceptional semi-conducting qualities and unusual thickness, 2D layered materials exhibit particular physical and chemical characteristics. In this respect, the ultra-thin geometry, outstanding electromechanical responsiveness, and other distinctive physical features of 2D piezoelectric nanomaterials have made them extremely attractive. Piezoelectricity is the capacity of a non-centrosymmetric material to produce polarization charges in response to the mechanical stress exerted from the outside. In addition, they are anticipated to be strong contenders and platforms for the creative design and advancement of future nanomechanical systems, self-adaptive nanoelectronics/optoelectronics, and intelligent robots. The well-known phenomena of piezoelectricity have been extensively researched both theoretically and experimentally. (Figure 2A). The same piezoelectric coefficient has been recorded in several instances with various values for each 2D material. In perspective of these variances, we compare the relaxed-ion piezoelectric coefficients derived from several references. The relationship between the piezoelectric coefficient (e) and the elastic stiffness characteristics of the material determines the piezoelectric coefficient (*d*), which evaluates the material’s mechanical to electrical energy conversion ratio. Figure 2B provides the piezoelectric coefficient *d*_11_ of well-known 2D transition metal dichalcogenide (TMD) materials. This suggests that the polarization of the ions changes as a result of the applied strain, which is the main source of the piezoelectricity of TMD monolayers.^3^ The intrinsic piezoelectric characteristics of TMDs have recently been explored (Figure 2C). Due to a substantial band offset brought on by the creation of the heterojunction, the piezoelectric response of the SnS_2_/SnS-based 2D thin film was ∼40% higher than that of the pure SnS_2_ thin film. The SnS_2_/SnS heterostructure can be used to create an adaptable energy harvesting device or an attachable, self-powered sensor for tracking heartbeat and movement in people.^4^ Figure 2D illustrates graphically how fixed charges may be held at the SiO_2_-air gap interface via tunneling triboelectrification and function as ghost floating gates by monolayer graphene on a SiO_2_ insulator with an atomic force microscopy (AFM) tip. Tunneling triboelectrification is almost exclusively only possible for 2D materials. The lower panel of Figure 2D demonstrates that as the number of graphene layers increases, the potential difference between the rubbed and unrubbed parts gets smaller until it completely disappears for highly oriented pyrolytic graphite (HOPG). The benefits of tunneling triboelectrification for piezo-semiconductive 2D materials may be essential, although they have only been reported so far for chemical vapor deposition (CVD) graphene. Graphene is naturally non-piezoelectric; however, researchers experimentally demonstrate piezoelectricity in graphene on SiO_2_ substrates (Figure 2E). As shown schematically, a chemical interaction between the carbon atoms of graphene and the oxygen atoms of the substrate caused a non-zero net dipole moment.^5^ In general, the conductivity of graphene is shape- and defect-dependent and should filter out piezoelectricity. On the other hand, porous graphene can function as a dielectric. Recently, studies revealed using the combination piezoelectric and triboelectric characteristics of 2D cobalt telluride (CoTe_2_) to produce power from waste heat (Figure 2F). The piezo-triboelectric nanogenerator generated an open-circuit voltage of around 5 V while exerting a force of 1 N and the influence of temperature in the range of 305–363 K, reveals a 4-fold increase in energy conversion efficiency (Figures 2G and 2H). In recent years, in addition to piezoelectric and triboelectric properties, flexoelectric property is also used to harvest energy from atomically thin 2D materials. Flexoelectric property appears as a polarization induced by the effect of strain gradients on crystal structures through curvature when applied using piezoelectric materials like h-BN, MoS_2_, Co_2_Te_3_, biotene (natural oxide), NbC, etc., and it has the potential to enhance its energy generation properties. Among them, graphitic-C_3_N_4_ does not have piezoelectricity. But the existence of the abnormal piezoelectricity in g-C_3_N_4_ is caused by flexoelectricity and the presence of non-centrosymmetric pores in the sheet.

**Figure 3:**
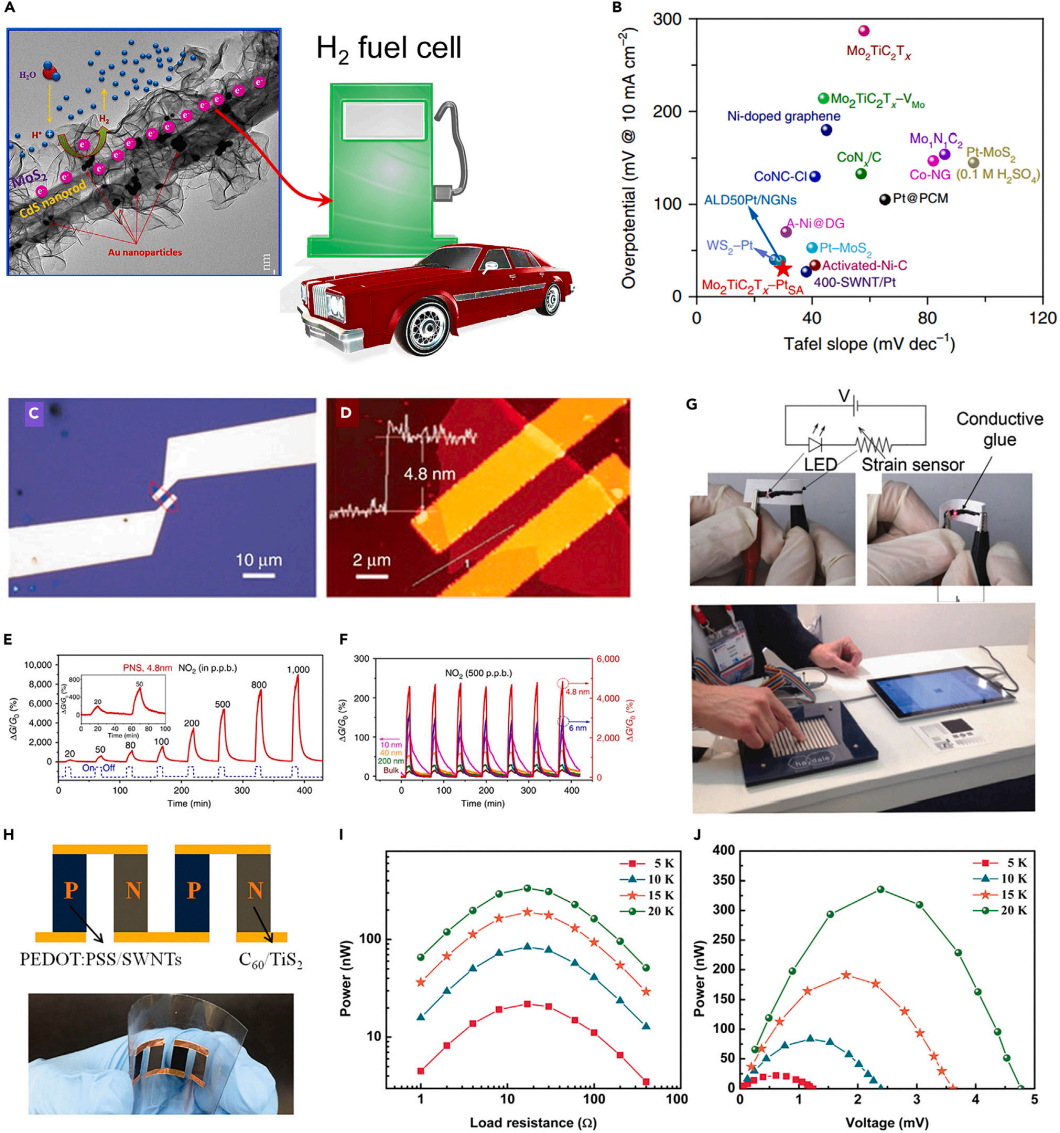
2D materials as a catalysts and sensors (A) TEM image demonstrating the development of a 1D CdS-Au/MoS_2_ hierarchical core/shell hetero nanostructure with increased photocatalytic H_2_ evolution.^10^ (reproduced with permission from American Chemical Society). (B) Comparison of Tafel slope and overpotential (10 mA cm^−2^) for different single-atom or Pt-based HER catalysts.^14^ (reproduced with permission from Nature). (C and D) The PNS sensor device’s optical and atomic force microscope pictures demonstrate how the PNS electrically connects the Au electrodes. (E) Relative conductance change dynamic response curves vs. time for NO_2_ concentrations ranging from 20 to 1,000 ppb. (F) The PNS sensor’s thickness-dependent multi-cycle responses to 500 ppb NO_2_^15^ (reproduced with permission from Nature). (G) Upper panel shows the diagram of a circuit that uses a graphene strain sensor as a variable resistor under compressive and tensile stresses (Lower panel).^16^ (reproduced with permission from Elsevier). (H) Photograph of flexible thermoelectric device.^17^ (I and J) Power generated as a function of load resistance and voltage with different temperature.^17^ (reproduced with permission from Royal Society of Chemistry).

Due to unique physical and chemical characteristics, including their higher surface area, abundance of active sites and active edges, increased conductivity, anti-photo corrosion, and chemical stability, 2D materials have been successfully used as catalysts, sensors, conductive ink, and in energy storage applications. For energy storage and conversion, these advantageous qualities are particularly helpful.^10^ Various examples of 2D nanosheet applications for energy conversion and storage are shown, including various nanosheet patterns utilized in supercapacitors, battery electrodes, electrocatalysis, and photocatalysis. Such nanosheet-based processes can be activated by both energy and light.^11^^,^^12^ Additionally, vacancies can provide charged sites that can trap electrons or holes to improve carrier separation in photocatalysis. The abundance of edges of nanosheets with coordination unsaturation and dangling bonds provide effective catalytic sites, which may be controlled to tune the electronic structure and related catalytic activity.^13^ There are a lot of edge sites in the MoS_2_ layers that are active sites for the hydrogen evolution process in the CdS-Au/MoS_2_ hybrid structures (Figure 3A). More crucially, the developed hybrid structure ensures the transport of photogenerated electrons from the CdS nanorods to the growing MoS_2_ nanosheets as well as the Au metal surface, thereby enhancing the electron-hole separation.^10^ This strategy can be a good choice for future fuels for transport.

Additionally, 2D materials are useful for measuring (theoretically and empirically) catalysis, such as hydrogen evolution reaction (HER), oxygen evolution reaction (OER), oxygen reduction reaction (ORR), etc. The most efficient catalyst for HER is the noble metal Pt, and even with lower catalyst loadings for precious metals like Pt, substantial activity may still be achieved. Recently, one of the original papers describing Pt single atom at the MoS_2_ surface continues to maintain the record for the lowest overpotential. Furthermore, double-transition metal MXene nanosheets with surface vacancies have been found to the lowest potential in acidic conditions (Figure 3B). This finding suggests that immobilized single Pt atoms on MXene can increase catalytic activity while significantly lowering the cost of the HER catalyst. The catalytic activity of several 2D materials has already been documented in a number of review publications and research articles. Therefore, we won’t have to get into more detail concerning 2D material catalysis in this study.

The prospects for device scaling are significantly impacted by the final thin body of 2D materials. The function of a sensing material is to interact with analytes and to adjust its internal physical characteristics in response to this contact. Tuning the surface chemistry of graphene materials is the most fundamental and straightforward way to change its sensing characteristics since the surface of graphene plays a vital role in intermolecular interactions. The same year, researchers used the “Scotch tape” mechanical exfoliation approach to insert 2D layers of black phosphorus (BP) onto Field-effect transistor (FET) devices, and they then used the resultant device for the sensitive detection of NO_2_ across a broad concentration range of 20–1000 ppb (Figures 3C–3E). The authors reported on the BP layers’ thickness-dependent sensitivity to a specific analyte (Figure 3F).

Researchers have also demonstrated the fabrication of printable 2D material applications and devices through the use of digital inkjet printing. In this context, adherence to the substrates might be quite difficult, for instance when making optoelectronic devices. This is because polymeric binders, which restrict the total functions of the 2D materials, cannot be used in the formulation of ink. As a result, there is a substantial obstacle to the increasing application of 2D material inks in the design of suitable binder systems for such applications. However, the currently available binder-free inks can still be put to fascinating uses in small-scale gadget demonstrations. A graphene strain sensor made using an inkjet on paper was recently presented by some group. An active strain detecting line is sandwiched between electrical contacts that were inkjet printed as shown by the schematic sensor setup in the inset of Figure 3F. The printed graphene strain sensors showed a rapid, noticeable change in electrical resistance under tensile and compressive forces as they were presented. The printed sensor was additionally used by the authors as a variable resistance in an electrical circuit to adjust an LED’s brightness when subjected to tensile or compressive strains. Pressure sensors can be made using structures in which an active sensing layer is “sandwiched” between contacts, in which the active sensing spots are connected to contacts or by combining the two (Figure 3F). Few research on printed 2D material pressure and touch sensors have been reported. However, commercial companies have used similar devices in a number of demonstrations. In this panel design, a printed electrode matrix is sandwiched between printed graphene/binder composite, allowing the matrix to sense resistance changes brought on by pressure and pinpoint the spot of contact.

Thermoelectric devices have attracted a broad investigation attraction owing to their ability to convert a temperature difference into electrical power and vice versa. Moreover, the thermoelectric properties of traditional inorganic and organic materials have significantly improved over the past few decades. Layered 2D materials, such as graphene, transition metal dichalcogenides, MXenes, black phosphorus, and IVA–VIA group compounds, have developed considerable research attention as a group of potentially high-performance thermoelectric materials. Their unique excellent transport properties and high-power factors make them promise and anticipated to be the next-generation high-performance thermoelectric materials. Thermoelectric materials based on Biand Sb could be attractive options for next-generation thermoelectrics due to their buckled and puckered structures. Thermal conductivity in Group V elements is anisotropic along specific preferred directions because of structural anisotropy. Antimonene,^18^ arsenene,^19^ and phosphorene^20^ are predicted to have a much lower thermal transport in the armchair direction compared to the zigzag direction. As with layered SnSe,^21^^,^^22^ anisotropic thermal transport in combination with buckled or puckered structures leads to very low thermal conductivities. Ab initio calculations, blending with the Boltzmann transport equation for phonons, demonstrate that antimonene has a low lattice thermal conductivity (15.1 W m^−1^ K^−1^ at 300 K), signifying its possible thermoelectric applications. In phosphorene, the calculated thermal conductivity along the armchair is 13Wm^−1^K^−1^, and in the zigzag direction, it is 30 Wm^−1^ K^−1^.^23^

These values of thermal conductivity are almost near to that of traditional bulk thermoelectric materials (typically <5 W m^−1^ K^−1^).^24^ Notably, changes in dimension from 3D to 2D result in changes in the Seebeck coefficient and electrical conductivity, which also affect the thermoelectric materials figure of merit (*ZT*) factor. In phosphorene^25^ and arsenene,^26^ first-principles calculations demonstrate that the electrical and thermal conductivities are orthogonal, thereby increasing the ratio between them, which enhances *ZT*. According to current theoretical studies, buckled antimonene has a *ZT* of approximately 2.1 at 300 K, while n- and p-doped bismuthene has a *ZT* of 2.1 and 2.4 at 300 K, respectively. Decoupling thermal and electronic contributions to *ZT* may enhance the thermoelectric figure of merit further with different morphologies such as nanoribbons.

Flexible thermoelectric materials have attracted significant attention because of their user-friendly and lightweight. Researchers explored 2D materials and fabricated a new class of flexible thermoelectric materials. Exfoliating TiS_2_ nanosheets from layered polycrystalline powders and assembling them with carbon nanocrystal (C60) nanoparticles resulted in the development of a new n-type flexible thermoelectric material by simultaneously increasing the power factor and decreasing the thermal conductivity (Figure 3H).^17^ Compared to the other solution-printable flexible n-type thermoelectric materials, C60/TiS_2_ hybrid films show a *ZT* ∼ 0.3 at 400 K. For large-area printing to make flexible thermoelectric devices, C60/TiS_2_ solution can be used as an ink (Figures 3I and 3J).

### Coating & painting using 2D materials

As a wettable ink or paint, most of the 2D materials are getting wider attention in the coating and painting field as it can be printed, penciled, or coated on various substrates for advanced applications.^27^ But the conductive properties of the coated 2D materials as well as the layered materials are not clear. There are several reports on the coating and painting applications of 2D material-based binary hybrid incorporated polymer materials, but the exact role of 2D materials in coating application should be analyzed in different aspects like barrier performance, functional anticorrosion behavior, and multipack-in-one synergism.^28^ Generally, the 2D materials are either prepared by exfoliation or solution-based method and in the end, the nanoparticles get suspended in a solvent or solvent mixture that can be further utilized as printable or coatable ink.^29^ Usually, the stability of the suspension can be explained by the help of Derjaguin-Landau-Verwey-Overbeek (DLVO) theory^30^ which suggests that after reaching an upper limit of concentration, the suspension will be no longer stable.^31^ Even though highly concentrated suspension is required for highly percolated networks, the rheological parameters like viscosity and rheology should also be monitored for the printing and coating kind of applications. As the particles in the suspension have the tendency to settle down, suspensions are considered as thermodynamically unstable in nature,^32^^,^^33^ hence the interaction between the solvent and the 2D materials should be enhanced so that the energy difference can be minimized.^34^^,^^35^ As a result of the particles’ tendency to settle out in such solvents, the dispersion medium is very constrained; therefore, different methods, such as combining it with binders, additives, and inks, can be used to improve the dispersion. Graphene ink’s viscosity is typically increased using cellulose acetate butyrate. But occasionally, these additions may have a negative impact on some desired features, necessitating their removal at the end of the formulation process. However, the cost of manufacturing goes up when these materials are removed through thermal processing.^28^^,^^36^

#### Anticorrosion property

Due to the damage it does to the environment and the associated financial loss, metal rust is seen as a severe concern. As a result, due to the physical barrier performance of organic coatings, boosting the corrosion resistant qualities is particularly important for extending the lifespan of metals. However, some of the volatile organic compounds (VOCs) employed as solvents could be harmful to human health and the environment. For the purpose of enhancing the performance of organic coatings, 2D materials such as boron nitride (BN),^37^ transition-metal carbides/nitrides (MXenes),^38^^,^^39^^,^^40^^,^^41^ and graphene^42^^,^^43^^,^^44^^,^^45^^,^^46^^,^^47^ are used as nanofiller. The highly ordered 2D structures of these materials function as preventative measures against coating flaws by creating tortuous paths for permeating water. Lewis’s acids and hydroxyl groups are present on MXenes (Ti_3_C_2_T_x_), which demonstrate that they assist the restricted movement of ions and water. Additionally, greater surface area is essential for improving the anticorrosive performance.^48^ In contrast to neat epoxy-coated metal, silk fibrin-Ti_3_C_2_Tx hybrids were used as a filler to improve the anticorrosive properties of water-borne epoxy. This is because they contain nanosheets of Ti_3_C_2_Tx that are capable of extending the diffusion channel of the corrosive medium, ensuring long-term protection. However, the study demonstrated that the aggregation of Ti_3_C_2_Tx nanosheets reduces the physical shielding, and under increasing hydrostatic pressure, protection is put at risk. However, the addition of silk fibrin to Ti_3_C_2_Tx improves the dispersion and interfacial interaction with epoxy, enhancing the material’s anticorrosive properties by generating a strong barrier layer. The surface modification slows down diffusion and remains functional even under intense pressure.^49^ For a short period of time, electrochemical experiments have shown that Gr coatings operate admirably as a barrier; nevertheless, for a long period of time in corrosive settings, the electrically conductive Gr may actually encourage metal corrosion rather than prevent it.^50^^,^^51^^,^^52^ Thus, insulator-modified graphene materials can be effectively used for anticorrosion applications.^53^ Being an insulating material, BN can be effectively used as filler for enhancing the anticorrosive properties owing to the long-term use. But the random distribution and orientation in an uncontrolled way leads to aggregation and will in turn affect the practical applications in a negative way.^54^^,^^55^^,^^56^ Consequently, surface functionalized BN were utilized and coated over the substrate by following electrodeposition strategy for ensuring order and uniform distribution.^57^ Additionally, it has been reported that, independent of graphene, a few layered TMDs, such as WS_2_ and MoS_2_, can exhibit superior anticorrosive properties because they reduce the formation of micro-galvanic coupling and create highly tortuous pathways for corrosive species.^58^ Due to graphene’s intrinsic barrier-shielding property and favorable electrical insulating behavior, fluorinated graphene (FrGO) has become a new material for the development of anticorrosive materials.^59^ Hence the fluorination of graphene is considered as a viable strategy for the mitigation of corrosive activity; but one particular issue has hindered the use of this material: it is difficult to adjust the degree of fluorination of the graphene via liquid phase exfoliation.^51^ For the synthesis of FrGO, the gas fluorination process can be successfully used, and the resulting FrGO will disperse well in the water-borne epoxy matrix. Although fluorination lowers electrical conductivity compared to GO, matrix agglomeration still presents a challenge. In order to improve the dispersive behavior, acridizinium ionic liquid (IL) modification can be successfully used. Furthermore, the lowest electrical conductivity of the FrGO helps in inhibiting the galvanic corrosion on the substrate material. The IL functionalization improves the compatibility with the epoxy matrix and thus ensures corrosion resistance of the composite coating.^60^ Figure 1B displays the anticorrosion mechanism taking place in fluorographene-based epoxy coating.

#### Superhydrophobic behavior of 2D materials

Superhydrophobic materials are receiving more attention in this century as a result of their cutting-edge use in numerous industries.^61^^,^^62^^,^^63^^,^^64^ Irrespective of physical-chemical properties, 2D materials are capable of showing advanced hydrophobic properties.^65^^,^^66^ Nature has traditionally served as a source of inspiration for science, and water droplets on lotus leaves have emerged as an important illustration of natural superhydrophobicity with contact angle >150°.^67^ Generally, the wettability of a surface is mainly determined by the composition and morphology of the substrate material. If surface roughness is high and the surface tension is lower, the material is said to be superhydrophobic.^68^^,^^69^^,^^70^ Wettable behavior and spreading rate of the 2D materials are affected by the peculiar atom organization in terms of holes and kinks, as well as the morphology.^66^ Nano composite materials with graphene coatings can be employed for deicing and anti-icing processes. The ability of graphene-coated superhydrophobic surfaces to clean themselves is its most appealing feature since water droplets may easily remove debris that has adhered to the surface.^71^^,^^72^^,^^73^ The potential for surface engineering graphene and its various forms for superhydrophobic coatings is still in its infancy, and this area of study is progressing quickly.^47^ Due to the pandemic epidemic Covid-19, superhydrophobic reusable and recyclable facial masks with high antibacterial and virucidal qualities are of utmost importance.^74^^,^^75^ Nano roughness created by carbon nanotube (CNT) forest particles over 3D graphene hybrids exhibit excellent superhydrophobic behavior over 2D graphene.^76^ MoS_2_ is also showing slight hydrophobic characteristics similar to graphene, and hence, it can also be utilized for high end superhydrophobic applications. Hence chemically inert superhydrophobic sponges made out of MoS_2_ is highly effective in showing better oil-water separation properties with good selectiveness and absorption ability toward oils and organic solvents.^77^

**Figure 4:**
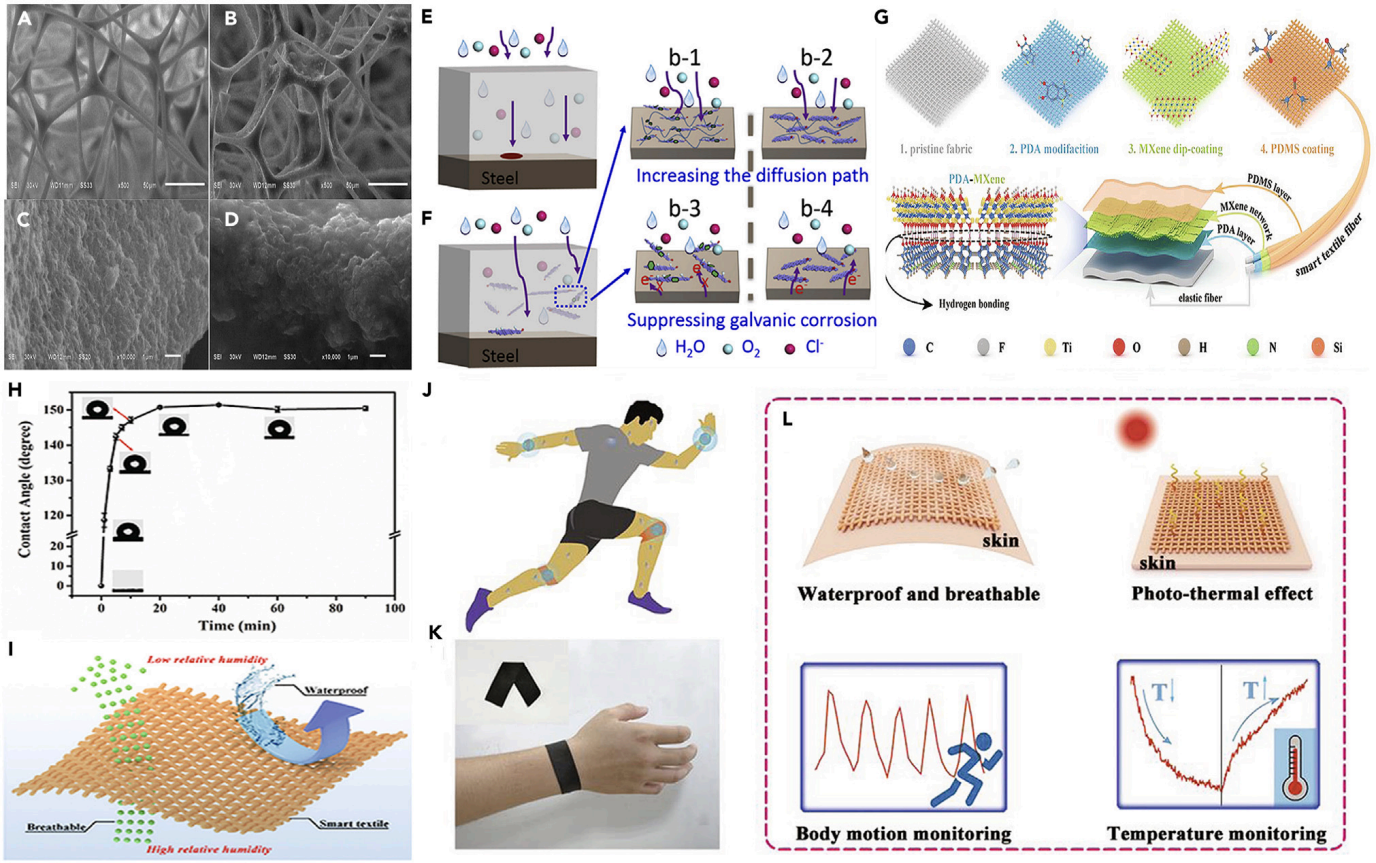
2D materials for superhydrophobic corrosion resistance (A–D)Typical SEM images of the pure melamine-formaldehyde sponge; SEM images of (B and C) MoS_2_ sponge and (D) MoS_2_@RTV sponge.^78^ (Reproduced with permission from Elsevier). (E and F) Illustration of corrosion protection mechanism for pure water-borne epoxy coating and fluorographene composite coatings respectively^60^ (reproduced with permission from Elsevier). (H) Water contact angles (WCAs) of the PM-3 textile as a function of dipping time in PDMS solution. (I) Schematic illustration of waterproof and breathable properties of the PM/PDMS textile. (G) Schematic illustration of the preparation process and structure of the PM/PDMS textile. (J) The PM/PDMS textile attached to the joints of the human body monitoring human motion. (K) The photograph exhibiting the textile as a smart strain sensing device worn on the wrist of the volunteer. (L) Its superb property and various promising applications. Reproduced with permission from Elsevier.^79^

But weak van der Waals force of interaction diminishes its practical applications owing to the shedding of MoS_2_ powders into the water or oil, which in turn causes contamination. Hence improving the stability of MoS_2_ in superhydrophobic sponges is considered as a research question. Super-oleophilic and superhydrophobic sponges made out of MoS_2_ and melamine-formaldehyde which made after modifying it with vulcanized (RTV) silicone rubber is considered as a remedy. Figure 4A shows the porous structure of MoS_2_. The highly porous and open pore-network structure helps in taking oil from water. The crater-like protrusions of the highly stacked MoS_2_ helps in forming highly roughened lotus leaf-like structures, these hierarchical nanostructures help in attaining interface, where the air get trapped in the grooves beneath the water which helps in attaining the superhydrophobicity.^78^ MXene sheets that are assembled onto the surface of polydopamine (PDA) (Figure 4G) and then protected with polydimethylsiloxane (PDMS) ensures superhydrophobic characteristics with anticorrosion ability (Figures 4H and 4I). These materials are thus utilized for the generation of smart textiles for wearable electronics application. Owing to the waterproof and breathable properties, photothermal and electro-thermal properties, these materials can be utilized for thermal sensing and strain sensing properties as shown in Figures 4J–4l.

### Optoelectronics applications of 2D materials

**Figure 5:**
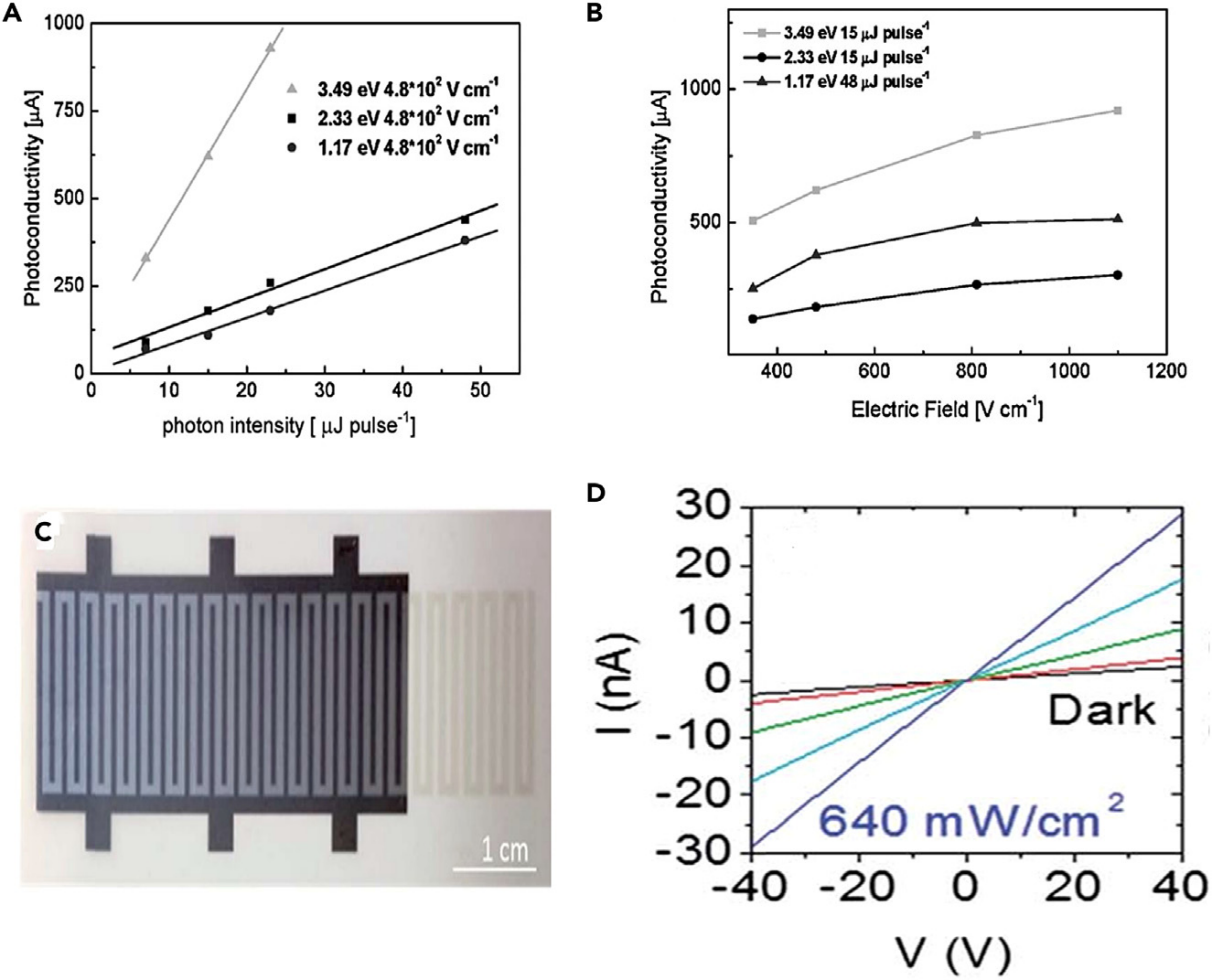
Optoelectronics applications of 2D materials (A and B) Incident light intensity and b) external electric field dependence of photoresponse with different photon energies for the drop-cast graphene film. Reproduced with permission from Wiley.^97^ (C and D) Photograph of printed photodetector and d) Current–voltage curves for the photodetector in the dark and under various illuminations up to 640 mW cm^-2^^32^ (reproduced with permission from Royal Society of Chemistry).

Graphene is a special substance with a zero-band gap that has a wide range of optical applications. Graphene and its related materials are capable of showing a broadband of response ranging from terahertz to UV spectral region. Semi-conductors often have strong optical response in the visible part of the spectrum whereas graphene has applications in the IR part of the spectrum. Moreover, the surface plasmon resonance by the graphene creates localized electric fields due to the in plane and lateral confinement effect.^80^ For biosensing application, stronger circular dichroism can be attained by enhancing surface plasmonic coupling in planar structured graphene.^81^ Che et al.^82^ studied the electrical modulating behavior and the capability to switch the absorption behavior of monolayer graphene owing to its optoelectronic applications. The magnetic dipole resonance in metamaterials can lead to a broadband absorption enhancement in near IR region. Photodetectors made of graphene are capable of showing surface plasmonic resonance and Fabry-Perot resonance which ensures broadband of absorption.^82^ It is possible to get several high-quality near-unity absorptions with multiplexing functionality, high modulation efficiency, and multi-functionalization of innovative optical devices as a result of multimode coupling of the metal-grating-assisted graphene plasmonic asymmetric system like graphene nanoribbon(GNR).^83^ 2D materials are capable of showing unbelievable characteristics through the exfoliation of layered structures. 2D materials-based semi-conductors generally possess n-type characteristics and rarely ambipolar characteristics. Thus, recent study revealed the development of P-type semi-conductors apart from doping strategy, 2D violet phosphorous were made with advanced optoelectronic features.^84^ Atomically thin TMDs with semi-conducting behavior are considered as next-generation material for optoelectronic applications.^85^^,^^86^^,^^87^^,^^88^^,^^89^^,^^90^ The strong optical absorption efficiency and ultra-fast charge transfer in TMDs like WS_2_, WSe_2,_ and MoS_2_ provides enormous applications in photodetectors and photodiodes.^91^^,^^92^^,^^93^ Compared to the chemically reduced graphene with defects, the liquid exfoliated few layered graphene has higher electrical conductivity and hence it ensures higher photo current collection and thus improves the operation speed of photodetectors. But as the few layer graphene (FLG) is highly pristine than rGO in terms of less functionalities and defects, thus the band gap will be almost zero, and thus photoresponse is very less owing to the ultra-fast recombination in femto seconds.^94^^,^^95^ Hence FLG is generally combined with polymeric materials, quantum dots etc.^96^ It is observed that the light intensity of rGO coated film depends on the photoresponse irrespective of the photon energies as shown in Figures 5A and 5B.^97^ Unlike single walled carbon nanotubes (SWCNT), which is having strong wavelength dependent spectra, GO generally respond to almost all the wavelength due to its wide absorption range^98^; Thus, rGO-based photodetectors generally show photo detection from UV^97^^,^^99^ to IR.^100^^,^^101^^,^^102^^,^^103^ In rGO-based photodetectors, it is generally observed that the photo current depends on the photo illumination position owing to the locally generated electric field via the Schottky barrier.^104^^,^^105^^,^^106^ Even though, there are photodetectors based on TMDs, compared to graphene it is still in its infancy stage.^107^^,^^108^^,^^109^TMDs are used in photo detecting applications either as single material^110^^,^^111^ or as polymer composites.^112^^,^^113^ Depending on the structure and electronic properties of chalcogenides and metals, TMDs can be classified into metallic, semi-conducting, insulating,^114^^,^^115^ and more importantly the band gap depends on the number of layers of dichalcogenides.^114^^,^^115^^,^^116^ MoS_2_ and graphene conductive ink is used for the fabrication of printed photodetectors (Figure 5C) that are capable of showing enfold increase in conductance compared to dark conductance during the laser illumination and the IV curves are shown in Figure 5D.^32^

### Construction and water purification

#### Composite materials in buildings

Ever rising demands of construction industry are met by updating existing materials and technologies. Construction cement currently used faces several limitations such as the need for repairs, thereby increasing the overall construction cost. To overcome the poor durability of current materials, new ones are extensively developed. 2D materials not only have the potential to overcome the durability, but also increase the strength of cement, thereby providing stronger structures. 2D materials have also been used for the development of specialty building materials with properties such as thermal and acoustic insulation, fire resistance etc, owing to their high strength, large surface area, ultra-thin sheet structures among other suitable properties. High demand for environment friendly building constructs has boosted the use of materials having a low carbon footprint.

Cement possesses properties such as compressive strength, durability, but may fail due to its brittle nature, relatively low tensile strength, and strain capacity.^117^ To overcome the problems, reinforcement fillers were incorporated into cement, ranging from steel bars to materials in nanoscale. Graphene and graphene derivative-based cement has found to expand concrete durability by improving resistance to environmental factors.^118^ Graphene can increase the durability of concrete by enhancing its mechanical and chemical properties. Paints containing graphene-based materials have anticorrosive and self-healing properties that promote crack and scratch resistance. Addition of these materials reduces repair cost and long-term durability of structures.

GO is hydrophilic due to the oxygenated functionalities attached to the aromatic ring, making the dispersion of GO in the cement matrix easier.^119^^,^^120^ This is advantageous to the otherwise costly process dispersing nanomaterials in dry cement. Flexible ceramic nanofibrous sponge-based composite of rGO in ceramic fibers were effective to control noise pollution.^121^ Another factor that would be affected by graphene-based concrete is the decrease of carbon emissions paving way for the development of clean and green concrete.

#### Specialty properties and biomimetics

**Figure 6:**
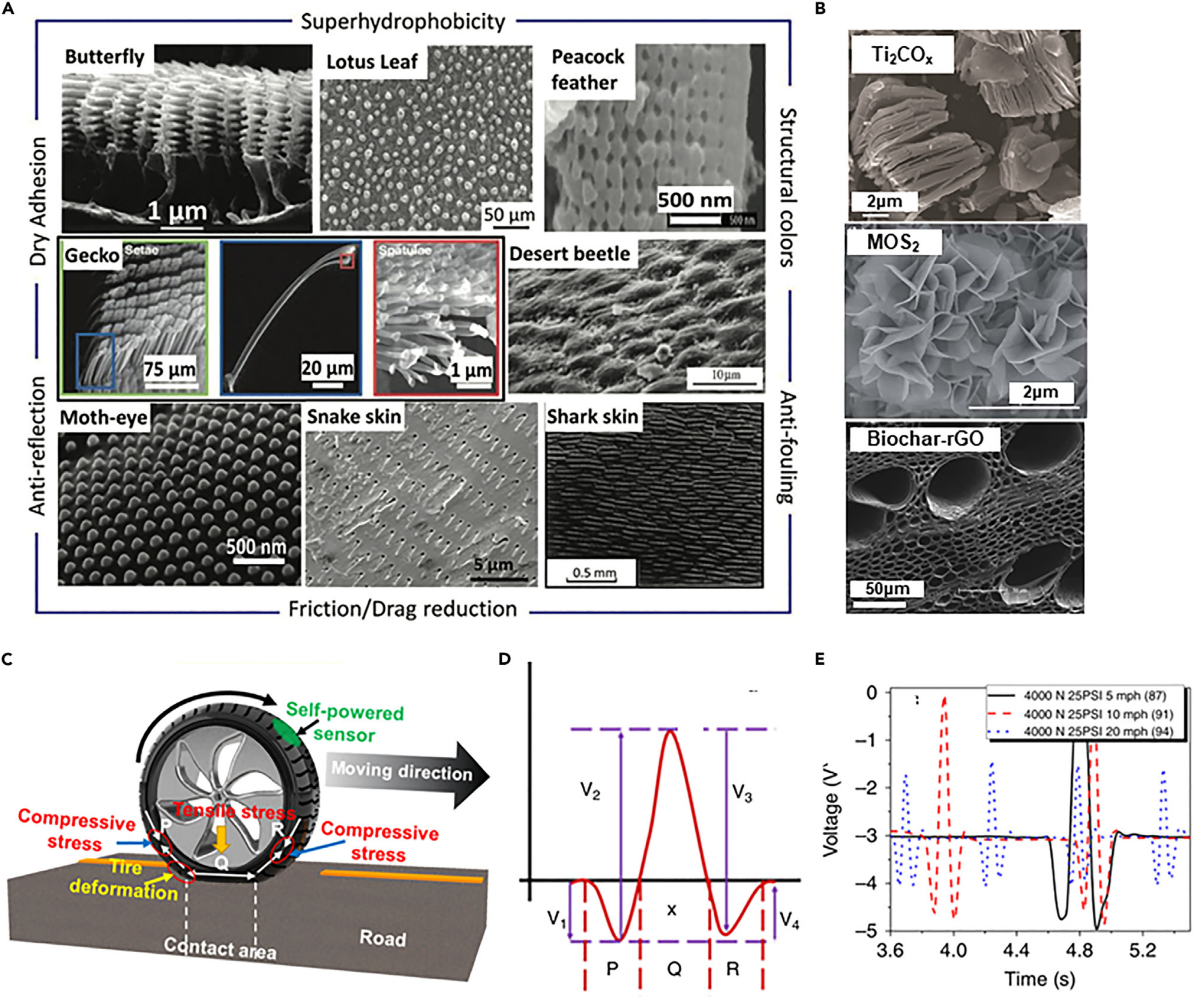
Biomimetic characteristics of 2D materials (A) Images of natural hierarchical surface structures with specialty properties.^128^ (B) Surface of 2D materials with the ability to mimic the natural structures, (top to bottom) Organ-like structure of Ti_2_CO_x_^129^ (reproduced with permission from Elsevier), MoS_2_ nano-petals^130^ (reproduced with permission from Royal Society of Chemistry) and layered porous structure of Biochar-rGO^131^ (reproduced with permission from Springer). (C and D) Schematic of 3D printed tire deformation with different types of strains and corresponding waveform. (E) Output across 3D printed sensor.^132^ (reproduced with permission from Nature).

Properties of natural materials (Figure 6A) due to hierarchical structural surfaces have been mimicked to develop surfaces with superior properties.^122^^,^^123^^,^^124^ Properties such as super hydrophobicity, anti-fouling, dry adhesion, anti-reflection, super-lubricity (zero friction) etc.^125^^,^^126^^,^^127^ can be incorporated into coatings and composites for synthesis of construction materials. The structural characteristics of 2D materials as those shown in Figure 6B has the capability to bio-mimic such properties due to inherent hierarchy. Wear resistance and flexibility are properties onlooked for the development of durable materials subjected to arduous conditions namely in aerospace, automobile parts, wind turbines, biomedical devices etc.

The self-healing behavior of graphene-based materials attributed to surface enhancement by stress transfer can prevent crack propagation and subsequent mechanical failures of coatings.^133^^,^^134^ This allows graphene to be used to enhance the properties of polymer matrices within coatings. Anti-wear properties of graphene originate from its hydrogen-bonded structural system.^135^ MXenes such as Ti_3_C_2_T*x* nanosheets, have shown drastic reduction in friction and wear at moderate stress and low humidity conditions.^136^ Ti_3_C_2_/nanodiamonds also demonstrated tribofilm features.^137^ Addition of MXene to Aluminum-based composite enhanced the mechanical properties to provide wear resistance.^137^

Several problems in the automobile industry can be overcome by utilizing the properties of 2D materials. They have shown good properties such as mechanical strength, good lubricity, and thermal stability, thus acting as promising coating candidates for aeronautics and space applications, where specialty properties as mentioned previously are required.^138^^,^^139^ A device with super capacitive properties consisting of nickel cobalt oxide reduced graphite oxide (NiCO_2_O_4_–rGO) composite showed good stability toward multi-cycle charging and discharging indicating its application in electric vehicles.^138^ 3D printing is a versatile technique for translating laboratory level constituents for use in the real world. Previous study combines 3D printed strain gauges, flexible piezoelectric energy harvester for powering the sensors.^132^ Graphene-based ink was used to print strain sensor capable of measuring tire-road interactions at varying driving speeds, normal load, and tire pressure. Figures 6C–6E shows the schematics of the tire deformation with different types of strain and corresponding waveform and the varying voltage output across the 3D printed piezoresistive sensor. This study can be expanded for the development of cost-effective smart tires with practical self-powering capability.

Membranes are a practical method by which air composition can be modified for on-board use. Implementation to vehicles depends on both technical and economic feasibility. 2D materials such as graphene oxide have already been shown to be successfully fabricated in membrane architecture to separate several different gas and liquid mixtures.^140^^,^^141^ These materials show the potential for development of selective air filters to reduce emission of gases that lead to environmental problems. The huge sorption and transport data available for graphene-derived materials help to guide the design of new 2D materials such as MXene, BN, MoS2, etc.^142^

## Biomedical applications of 2D materials

### Drug delivery

The requirements of an efficient drug delivery system (DDS) include high drug loading rate, good biological distribution, and low toxicity.^143^^,^^144^ The physical, chemical, and structural properties of 2D materials make them potential nanoscale drug delivery systems. Mainly, the large surface area due to ultra-thin nanosheets and response to stimuli at physiological conditions make them ideal DDSs. Near infra-red (NIR) rays show adequate penetration through tissue, making it suitable to control drug release rates. 2D materials show response to NIR radiations make them suitable for photothermal therapies for controlled drug release in wound healing, cancer diagnostics, and therapy etc.

**Figure 7:**
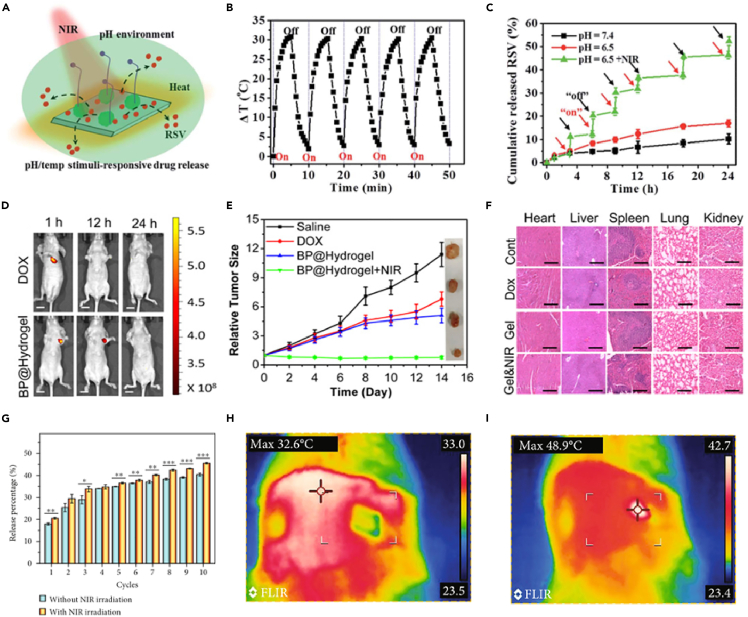
Use of 2D nanomaterials for efficient drug delivery (A) Schematic illustration of pH/temperature stimuli-responsive drug release of utReS2@RSV–FA. (B) The thermal stability of utReS2@RSV–FA indicated by experiments subjecting it to 5 cycles of irradiations (5 min, 808 nm, 1 W cm^2^). (C) Effect of NIR laser irradiation on the release kinetics of RSV from utReS2@RSV–FA at pH 7.4 and 6.5.^148^ (reproduced with permission from Royal Society of Chemistry) *In vivo* study of effect of black phosphorous hydrogel on tumor in mice. (D) Fluorescence images of mice at different time intervals after *in vivo* photothermal assay (Scale bars: 1 cm). (D) Tumor growth curves of mice treated with hydrogel with and without irradiation. (F) H&E-stained images of major organs from treated mice. (Scale bars: 50 μm).^144^ (reproduced with permission from Royal Society of Chemistry). (G) Photothermal test of MXene-integrated hydrogel and release profile of adenosine from PBA-integrated hydrogel with/without 10 cycles of NIR irradiation. (H and I) Thermal images of the microneedle of the patch on rat skin (h) before and (i) after NIR irradiation.^149^

Properties of graphene and graphene derivatives such as the presence of delocalized surface π electrons and large surface area allows loading of poorly soluble drugs.^145^^,^^146^ GNRs^147^ showed doxorubicin (DOX) loading capacity as high as 235% by weight. Polyethyleneimine-grafted GNRs were found to be useful in gene therapy owing to their ability to recognize low toxic microRNA via cellular delivery. TMD consisting of ultra-thin rhenium disulphide nanosheets (utReS_2_) modified with resveratrol (RSV) conjugated with folic acid (FA) formed a nanocomposite utReS_2_@RSV–FA that demonstrated potential as a drug delivery nano-platform for cancer therapy.^148^ Figure 7A shows the schematic illustration of dual (pH/temperature)-stimuli-responsive release of RSV. Results, shown in Figures 7B and 7C, confirm the materials capability to show dual-stimuli controlled drug release potential with photothermal stability in tumor (slightly acidic pH) environment.

An important aspect to be considered for the development of DDSs is the biodegradability of the materials. Though many polymer-based hydrogels are being widely used in biomedical applications,^150^^,^^151^^,^^152^ 2D materials are still being explored. One such biodegradable temperature-sensitive hydrogel (gelation at body temperature) was developed by combining agarose and PEGylated black phosphorous nanosheets (BPNs).^144^ The hydrogel showed NIR-light controlled release of DOX. Both *in vivo* (Figures 7D–7F) and *in vitro* results concluded that this hydrogel-based drug delivery platform with low cytotoxicity and high biodegradability has great potential for clinical therapeutics of breast and melanoma cancers. MXenes capacity to convert light into thermal energy via NIR irradiation, has facilitated its utilization for NIR stimulated DDSs. Microneedle patches mimicking micro well template was developed using a boronated and MXene-mixed hydrogel for injury repair.^149^ Light activation of MXene triggered the release of adenosine, thereby promoting angiogenesis speeding the wound healing as shown in Figures 7G–7I.

### Scaffold and tissue engineering

Tissue engineering (TE) is the regeneration and regrowth of tissues and organs that are damaged by trauma or birth defects. To facilitate effective regeneration, the engineered tissues require cellular organization that resembles that of normal cells. More challenging bone TE requires restoration of bone defects by conferring osteo differentiation or osteo-inductive capability on scaffold materials. For successful bone TE, a highly functional scaffold is inevitable. Properties such as biocompatibility, biodegradability, enhanced cell differentiation and proliferation, and support are crucial factors to be considered for selecting a suitable scaffold material. As discussed in the previous section, 2D materials have shown prospects in several biomedical applications such as TE, owing to their nanoscale structural features and suitable electronic, optical, and biological properties.

Studies show that graphene and its derivatives have been found to improve the biological properties of scaffold materials, due to their ability to promote adhesion and proliferation of cells.^153^ Their ability to enhance the growth of osteoblast cells (osteogenesis) has been utilized in bone TE. In addition, providing good mechanical strength is essential for tissue development. Nanocomposite composed of GO and polyvinyl alcohol (PVA) was fabricated by selective laser sintering (SLS) for bone TE.^154^ The mechanical properties in terms of compressive strength, Young’s modulus and tensile strength were improved. Human osteoblast-like MG-63 cell growth was enhanced by the scaffold, indicating good cytocompatibility. GO nanoflakes incorporated into a matrix consisting of gelatin and hydroxyapatite, showed good mechanical strength and osteogenic differentiation.^155^ rGO-hydroxyapatite composite showed osteogenesis of MC3T3-E1 pre-osteoblasts, thereby promoting bone regeneration. This material showed promising results in dental and orthopedic bone regeneration.^156^

Titanium carbide (Ti_3_C_2_) was combined with MXene nanofibers to form nanocomposites for TE and cell culture applications as the functional groups on the composite promote cellular growth.^157^ To study the activity of the nanofiber, mesenchymal stem cells (BMSCs) derived from bone marrow were analyzed. The nanocomposite fibers showed cell differentiation and proliferation. As discussed in the previous section, (NIR)-triggered photothermal activity of MXene finds several applications in tumor therapy.^158^ The nanocomposite showed improvement in growth of tissue within bones with the ability to destroy bone tumor cells. When compared to graphene, MXenes hydrophilic nature makes it suitable for combining with polymers.^159^^,^^160^ Such advanced properties enable its use in developing biocompatible materials for localized therapy. One such example is the development of a soluble microneedle system, composed of 2D MXene and polyvinylpyrrolidone with photothermal activity for skin penetration.

Injectable hydrogel-based materials are particularly useful in bone TE that requires repair of irregular tissues. Such an injectable gel was developed by combining injectable CNT and BP gel.^161^ The material showed enhancement of osteogenesis of MC3T3 pre-osteoblast cells due to the presence of BP that aided the release of phosphate ions. The results suggested that the hydrogel enhanced adhesion, proliferation, and osteogenic differentiation of the cell, thereby showing promising potential for future bone TE.

### Diagnostic devices and imaging

Several diagnostic techniques currently used are unsuitable for cancer detection at initial stages. Also, techniques such as surgical resection may lead to patient trauma. Due to high cost and invasive nature of existing techniques, there is a high demand for development of reliable biomarkers with high specificity. 2D materials can serve as a versatile platform for binding biomarkers for detection and treatment of diseases. Aptamers such as DNA and RNA can be easily bound to GO with large surface area.^162^ The binding can be pH controlled, thereby controlling drug delivery in acidic tumor physiological environment facilitating a targeted delivery of chemotherapeutic drugs. These findings not only promise easy diagnosis by delivery of imaging agents, but also control toxicity and reduce side effects.

**Figure 8:**
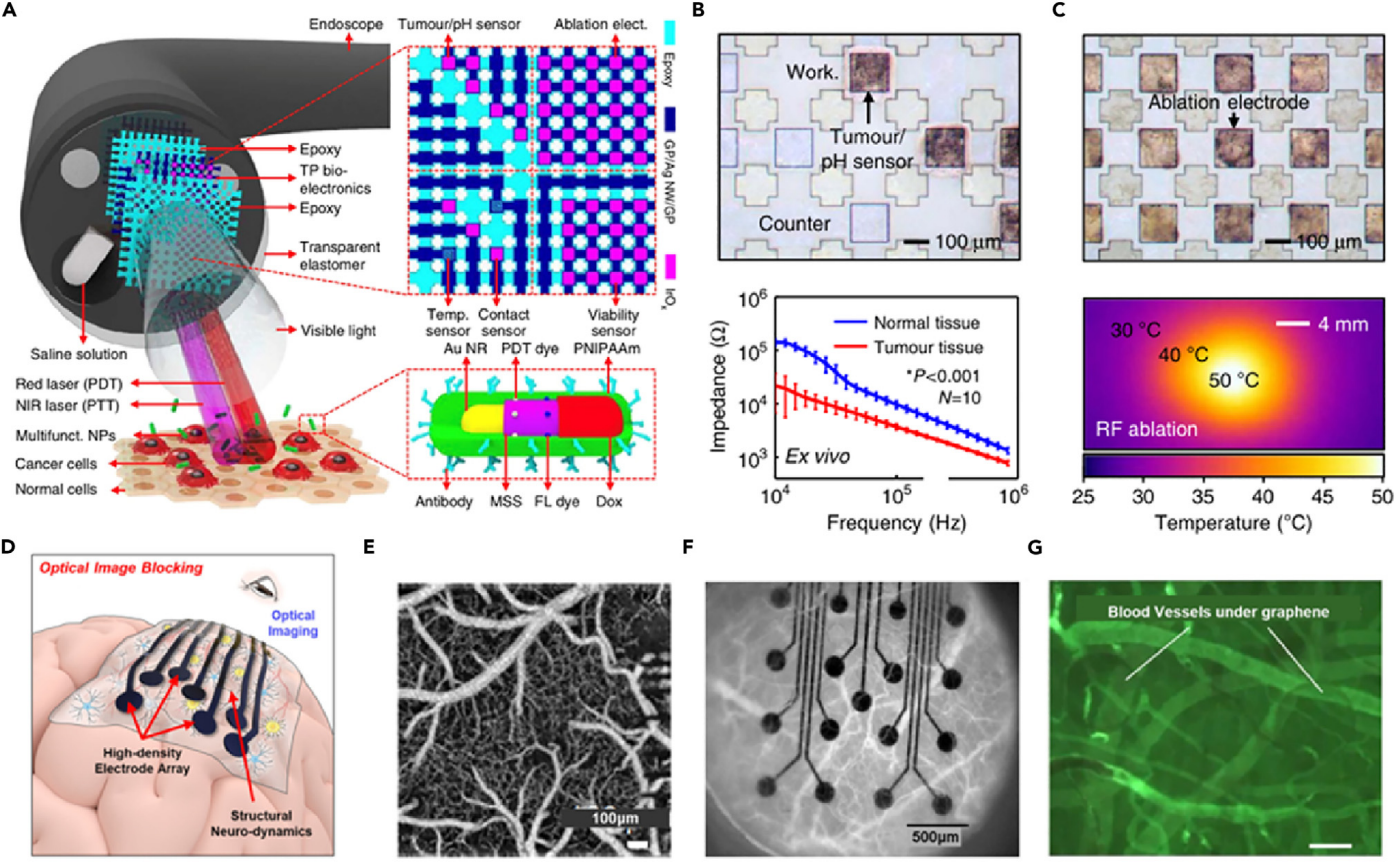
Bioelectronic devices and therapeutic devices based on 2D materials (A and B) Functional endoscope based on transparent bioelectronic devices and theragnostic nanoparticles (A) Schematic illustrations of the design strategy and mode of use using (B) Optical microscope image of tumor and pH sensor (top) and *ex vivo* comparison of impedance of tumor (HT-29) and normal tissues *ex vivo* using graphene electrode (bottom). (C) Optical microscope image of ablation electrode (top) and the infra-red camera image during the Radio frequency ablation (bottom).^164^ (D) Schematic image of optical image blocking from non-transparent electrode array with integration of optical modalities. (E) Optical coherence tomography (OCT) imaging of a brain with a transparent graphene electrode array. (F) An image of blood vessels from the brain cortex labeled with rhodamine-B dextran through the platinum electrode array from the cranial window. (G) Fluorescence image of blood vessels with a transparent graphene electrode array.^165^ (reproduced with permission from Nature).

Another challenging diagnostic target is the gastrointestinal tract due to the tedious enterology procedures targeting polyps and cancers accompanied by profuse bleeding. Advancement in endoscopic diagnosis conjugates both the imaging and therapeutic procedures. However, due to limitations in current endoscopic techniques, there is a need for integration with nanoelectronics. Figure 8A demonstrates the schematic illustration of design strategy and mode of use for the multifunctional endoscope system that integrates bioelectronics with the aid of theragnostic nanoparticles. A transparent bioelectronic grid, constituting graphene prepared by chemical vapor deposition, doped with gold,^163^ and deposited with iridium oxide was used.^164^ Tumor detection by pH ablation sensors (Figure 8B), was able to differentiate the tumor tissues from normal ones according to impedance differences. pH levels measured by open-circuit potential serves as another diagnostic biomarker owing to the difference in pH levels around tumor tissues. Another alternative to surgery is radio frequency ablation and photothermal therapy. Figure 8C shows the radio frequency ablation characteristics.

Whispering-gallery mode (WGM) is a micro-laser technique useful for biological sensing that reduces the gap between laboratory research and day-to-to problems.^166^^,^^167^ Biomarkers such as DNA and proteins can be detected using WGM.^167^ Carbon-based materials show prospects owing to their biocompatibility. Graphene-based WGM lasers showed resonance in submicron range.^168^ Tungsten disulphide sandwiched between hexagonal BN were also utilized.^169^ MXenes show enhanced biosensing, due to their hydrophilic surface, large specific surface, and opto-absorption.^170^ Previous studies demonstrated the sensing ability of MXene making them suitable for detection of many biomarkers.

Flexible neural implantable systems are used for diagnosis and treatment of neurological diseases such as Parkinson’s disease, epilepsy, depression etc. There are several challenges with the integration of electrophysiological and optical modalities by non-transparent neural interfaces such as photoelectric artifacts, optical image blocking (Figure 8D), and light transmission efficiency.^165^ Figures 8E–8G shows the optical coherence tomography (OCT) imaging of a brain using a transparent graphene electrode array, image of blood vessels from the brain cortex labeled with rhodamine-B dextran, and fluorescence image of blood vessels with a transparent graphene electrode array in order. Good electrical conductivity and transparency to UV and IR light allows the use of graphene in neural interfaces.^171^ Graphene has good chemical and mechanical stability and flexibility required for such flexible bioelectronic applications.^172^^,^^173^^,^^174^ Calcium imaging is a useful approach in neural mapping capable of single-cell spatial neural resolution.^173^ Nitric acid-doped graphene electrodes were found to be useful for calcium imaging enabling electrophysiological recording and optical imaging. Graphene-based carbon layered electrode array^175^ was found to be applicable in diagnostics via fluorescence microscopy and 3D optical tomography.^176^

### Biosensing

In the field of biochemical sensing, 2D materials are promising over their bulk form due to their ability to provide high density of active surfaces over a large surface.^177^^,^^178^ Their ability to exhibit a broad range of electronic and optical properties, along with potential for functionalization can also be utilized in biosensing and healthcare applications.^179^^,^^180^^,^^181^ 2D material properties have been reported to have remarkably improved the sensitivity and efficiency of biosensors.^182^^,^^183^

Wearable biosensors are the most widely sought real-time method of sensing in which the data analyzed can be transmitted to a remote mobile device, such as a smartphone. An MXene/Prussian blue (Ti_3_C_2_T_*x*_/PB) multifunctional wearable biosensor was developed for sweat-based detection of biomarkers such as glucose and lactate.^184^ The biosensing ability of the wearable patch was demonstrated by monitoring sweat during 10–30 min of intense cycling activity. The results showed a correlation between physical exercise and lactate generation after a time delay. The sweat glucose measured showed agreement with the reported blood glucose levels.

FET based on 2D materials has huge potential for label-free biosensing due to their electronic properties. Zika virus epidemic has been a growing concern for public health. A graphene-based Zika virus biosensor was developed by interpretation of Zika virus antibody (IgM). The Agile R100 biosensor chip functionalized with anti-Zika NS1 provides a promising base for development into Zika clinical applications and improved diagnostic testing early in infection.^185^ FET based on semi-conducting TMD was used to develop a sensor by functionalizing tungsten diselenide (WSe_2_) monolayers for rapid and sensitive detection of severe acute respiratory syndrome coronavirus 2 (SARS-CoV-2).^186^ The device’s selectivity using bovine serum albumin (BSA) protein as the test molecule showed negligible response to BSA compared to the SARS-CoV-2 spike antigen protein.

### Anti-viral coating with 2D materials

**Figure 9:**
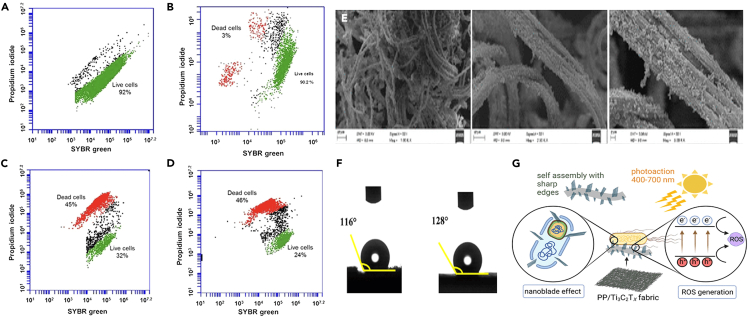
Antibacterial activity and mechanism of 2D materials (A–D) Cell viability measurement of *E. coli* and *B. subtilis* exposed to polyvinylidene difluoride (PVDF) and Ti_3_C_2_T_x_. Flow cytometry dot plots of *E. coli* (A and B) and *B. subtilis* (C and D) bacterial cells exposed to control PVDF (a and b) and MXene films (C and D) for 24 h reproduced with permission from Nature.^189^ (E) SEM image of MoS_2_-coated lotus fiber,^190^ (reproduced with permission from American Chemical Society). (F and G) Contact angle of pure and MoS_2_-coated fiber and (G) Schematic diagram of antibacterial activity in PP/Ti_3_C_2_T_x_ nanocomposites showing the synergistic physical nanoblade effect and ROS generation ^191^ (reproduced with permission from American Chemical Society).

Owing to the antibacterial properties, 2D materials like GO, MXene, and h-BN and its surface modified forms have gained wider attention. A recent study by Mazinani et al.^187^ proved the highest antibacterial property of GO and MXene over h-BN against *S. aureus* colonies with an activity of 70 (±2) % and 97 (±0.5) % respectively. They have also suggested that compared to film making, drop casting method is efficient for addressing both osseointegration and antibacterial abilities. The strong interaction of the 2D materials in the biological medium through membrane disruption and direct edge contact are beneficial in making 2D materials-based coatings in decreasing the adhesion and improving the cell destruction. But in the mammalian cells, it is very active enough to promote cell differentiation and tissue growth via better adhesion behavior, and hence, designing of coatings for cell specified action is highly advantageous.^188^ The dotted plots shown in Figures 9A–9D shows the populations of stained cells of *E. coli* and *B. subtilis* after 24 h of exposure to Ti_3_C_2_T_x_-coated membranes via flow cytometry. The strong fluorescence indicates the live bacteria and light fluorescence region indicates the dead bacteria, it should also be noted that after 24 h of exposure, the population changed from viable to dead or compromised cells. Compared to uncoated membranes, Ti_3_C_2_T_x_-coated membranes cause irreparable damage causing growth inhibition.^189^ Cellulose-based threads derived from Indian lotus when embroidered with MoS_2_, it is observed that the enriched the hydrophobic and antibacterial activity. The twisted ribbon like structure of the material (Figure 9E) helps in enhancing the permeability and moisture absorption. MoS_2_ coating on the fiber helps in enhancing the superhydrophobic behavior as shown in Figure 9F.^190^ In general, nanoblade effect is observed in composites with 2D materials like MXene as shown in Figure 9G, where the stress is mediated by the contact of highly sharp edges. Moreover, the ROS (Reactive Oxygen Species) effect coupled with MXene also plays a vital role in enhancing the antimicrobial activity.^191^

## Limitations

Owing to the tunability and ability to capture biomarkers, 2D materials find applications in the biomedical field. However, the toxicity of 2D materials remains a concern even though effective reduction in toxicity has been achieved with functionalization. Further research that focuses on the toxicity of 2D materials needs to be conducted. Graphene and other 2D materials are getting wider attention in different fields owing to the exceptional properties and can be employed for a myriad of applications as discussed in this review. Since 2D materials-based conductive ink has been studied for many years, it has become clear that efficient exfoliation in the required solvents is crucial for better dispersion and the preservation of solubility. As a result of their considerable adaptability, solution-based approaches can be further scaled up for industrial use.

## Conclusions and future perspectives

The paper reviews the recent progress of 2D materials in the field of energy harvesting, coatings, biomedical, construction, and automobiles. As mentioned throughout, 2D materials have innumerous opportunities for expansion in everyday domains. As highlighted in the paper, structural properties such large surface area, hydrophilicity, and hierarchy allow 2D materials to be combined with metals, ceramics, polymers etc., increasing their product value. Proper functionalization can lead to the development of products with tribological characteristics. Despite high prospects, research on 2D materials that deliver a fruitful outcome is still at its infancy.

In the construction sector, 2D materials have high potential for enhancing properties and reducing the carbon footprint. Scaling up production in bulk needs to be prioritized by major industries. The automobile industry is always in need of technologies that can overcome the huge energy demand and reduce the environmental impact. Extensive research on batteries based on 2D materials will extend their clean application in automobile sector.

In future, as a remarkably fast-growing field of nanotechnology, 2D material-based products have bright prospects and need further exploration. Biomimetic research with emphasis on the structural hierarchy, from micro-to macro-scales needs to be explored. For the development of specialty products, further optimization of 2D material properties and its combination with other materials are essential. Ultimately, the goal of research in 2D materials would be to develop materials for multipurpose use in industrial and everyday activities.

The addition of conductive polymers into the 2D-based ink helps in enhancing the conductivity and coating of 2D materials-based conductive ink over the polymeric flexible substrates can be further extended toward the energy harvesting applications. Highly conductive ink can also be incorporated into other polymers like epoxy, polyethylene, polypropylene, rubber etc. for making polymer composites which can be utilized for EMI shielding and anti-static applications which are having higher demand in automobile industry. Advances in large-scale 2D material growth, heterostructure synthesis, optoelectronic device fabrication, and integration technologies are projected to soon lead to real-world applications. Due to the financial loss brought on by the deterioration of machinery, pipelines, and monuments, anticorrosive coatings manufactured from 2D materials are particularly better for protecting steels, which is highly essential in industrial applications. In the event of pandemics like Covid-19, 2D materials-based anti-viral coatings could be extensively used in the biomedical field in the form of protective textiles, masks, gloves, etc. Therefore, the distance from lab to factory for 2D materials is very short, as evidenced by emerging innovation hubs based on graphene and other 2D materials in different regions of the world.

### Data availability

This paper analyzes existing, publicly available articles from the Scopus database. Any additional information required to reanalyze the data reported in this paper is available from the lead contact upon reasonable request.
